# Who benefit from school doctors’ health checks: a prospective study of a screening method

**DOI:** 10.1186/s12913-018-3295-3

**Published:** 2018-06-27

**Authors:** Kirsi Nikander, Silja Kosola, Minna Kaila, Elina Hermanson

**Affiliations:** 1 School and Student Healthcare, Department of Social Services and Healthcare, City of Helsinki, Finland; 2 Doctoral School in Health Sciences, Doctoral Program in Population Health, University of Helsinki, Helsinki, Finland; 3 Medical Services for Children and Adolescents, Department of Social Services and Healthcare, City of Helsinki, Finland; 4 Helsinki University Hospital, Children’s Hospital and University of Helsinki, Helsinki, Finland; 5 Public Health Medicine, University of Helsinki and Helsinki University Hospital, Helsinki, Finland; 6 Pikkujätti Medical Centre for Children and Youth, Helsinki, Finland

**Keywords:** Children, Student, Questionnaires, Screening, Health check, School health services

## Abstract

**Background:**

School health services provide an excellent opportunity for the detection and treatment of children at risk of later health problems. However, the optimal use of school doctors’ skills and expertise remains unknown. Furthermore, no validated method for screening children for school doctors’ assessments exists. The aims of the study are 1) to evaluate the benefits or harm of school doctors’ routine health checks in primary school grades 1 and 5 (at ages 7 and 11) and 2) to explore whether some of the school doctors’ routine health checks can be omitted using study questionnaires.

**Methods:**

This is a prospective, multicenter observational study conducted in four urban municipalities in Southern Finland by comparing the need for a school doctor’s assessment to the benefit gained from it. We will recruit a random sample of 1050 children from 21 schools from primary school grades 1 and 5. Before the school doctor’s health check, parents, nurses and teachers fill a study questionnaire to identify any potential concerns about each child. Doctors, blinded to the questionnaire responses, complete an electronic report after the appointment, including given instructions and follow-up plans. The child, parent, doctor and researchers assess the benefit of the health check. The researchers compare the need for a doctor’s appointment to the benefit gained from it. At one year after the health check, we will analyze the implementation of the doctors’ interventions and follow-up plans.

**Discussion:**

The study will increase our knowledge of the benefits of school doctors’ routine health checks and assess the developed screening method. We hypothesize that targeting the health checks to the children in greatest need would increase the quality of school health services.

**Trial registration:**

ClinicalTrials.gov Identifier: NCT03178331, date of registration June 6 ^th^ 2017.

**Electronic supplementary material:**

The online version of this article (10.1186/s12913-018-3295-3) contains supplementary material, which is available to authorized users.

## Background

School health services provide an excellent opportunity for the detection and treatment of children and young people at risk of later health problems [1]. Globally, the organization, financing and the functions of school health services vary significantly [2, 3]. According to a recent systematic review, health promotion interventions in schools have been effective in decreasing body mass index, increasing physical activity levels, and decreasing experiences of being bullied [4].

The optimal use of the school doctors’ skills and expertise remains unknown. A systematic review in the UK demonstrated that evidence is insufficient to assess the effectiveness of either the routine or selective school entry medical examination [5]. In a study from the Netherlands, doctor’s assistants carried out pre-assessments instead of all children being screened by a nurse or a doctor. This triage approach resulted in lower referral rates [6] and a cost reduction of about one-third [7] compared with usual practice. Overweight, visual disorders and psychosocial problems were detected similarly in both groups [8].

**Fig. 1:**
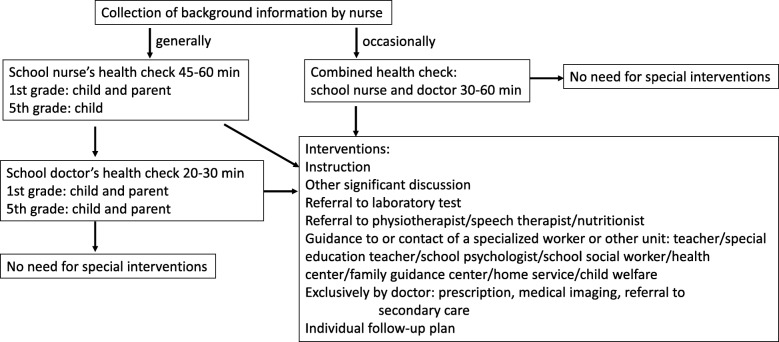
Current extensive health check in primary school grades 1 and 5 in Finland

According to Finnish law, the public preventive health care system of children between ages 0–6 years (Well Child Clinic) offers at least 15 routine health checks by the nurse and 5 health checks by the doctor, free of charge. School nurses, trained in health promotion and preventive care, arrange annual health checks in all grades of primary school. The health checks in grades 1, 5 and 8 (at ages 7, 11 and 14 years, respectively) are called “extensive health checks”. The parents are invited to participate. Both the school nurse and the school doctor assess the well-being of all children and young people in those grades (Fig. 1). Table 1 describes the division of labor in primary school grades 1–6. Most of the school doctor resources in primary schools are allocated to “extensive health checks” regardless of previously identified health risks. Children in other grades receive only very limited school doctor services although the special needs of all children should be recognized and help provided timely. When a special need is recognized and more time required to treat it, some of the school doctor’s obligatory health checks may be omitted randomly or the family may be instructed to contact the public health center or the private sector for help. The overall efficiency or the cost effectiveness of the Finnish school healthcare system has not been evaluated thus far. Currently, no validated method exists for screening children for school doctors’ assessments.

**Table 1:** Division of labor in school health services in primary school grades 1–6 in Finland^a^

Tasks	School nurse	School doctor
Extensive health examinations in grades 1 and 5	x	x
Collection of background information for the extensive health examinations	x	
Well Child Clinic (pregnancy and birth, biopsychosocial development)		
THL^b^ questionnaire by parent (1st and 5th grade), child (5th grade) and teacher (some schools)		
Statements from the student welfare group		
Evaluation: growth, vision, hearing, blood pressure, posture	x	
Complementation of background information		x
Evaluation of growth, somatic, psychiatric and neurologic status		x
Diagnostics and differential diagnostics		x
Vaccinations	x	
Referrals to physiotherapist, speech therapist, nutritionist	x	x
Referrals to secondary care		x
Guidance to or contact of specialized workers/other units	x	x
Teacher/special education teacher/school psychologist/school social worker		
Health center		
Family guidance center/Social worker/Home service		
Child welfare		
Referrals to laboratory tests	x	x
Referrals to medical imaging		x
Annual health checks (the general wellbeing, growth, eating, exercise and sleeping habits, friendships and hobbies)	x	
Prescriptions		x
Health education and support	x	x
Evaluation of special needs in all grades	x	x
Control visits	x	x
Participation in student welfare groups^c^	x	x

^a^Local variations may exist

^b^*THL* The National Institute for Health and Welfare

^c^Evaluate and develop the well-being of school community and students (permanent members: school principal, special education teacher, school psychologist, school social worker, school nurse)

The primary aims of this study are 1) to evaluate the potential benefits or harm of school doctors’ routine health checks in primary school grades 1 and 5 (at ages 7 and 11 years, respectively and 2) to explore whether some of the school doctors’ routine health checks in those grades can be omitted using study questionnaires that assess the parents’, school nurses’, and teachers’ potential concerns regarding each child. The secondary aim is to evaluate the implementation of the school doctors’ interventions and follow-up plans at one year after the health check.

## Methods

### Study design

**Fig. 2:**
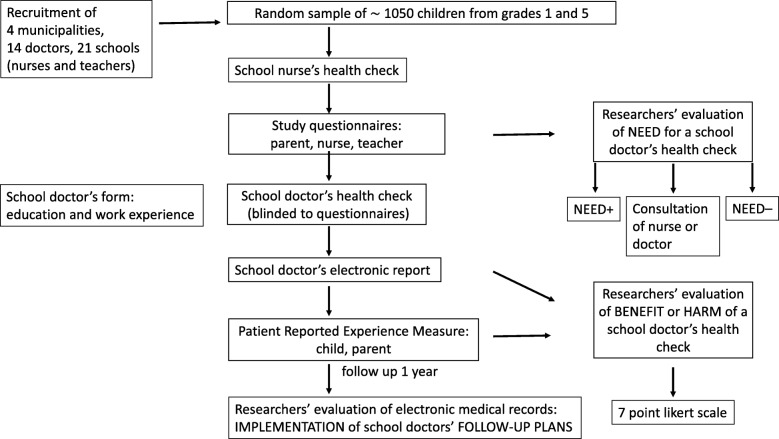
Study design

This is a prospective, observational, multicenter study which is conducted by comparing the need for a doctor’s appointment based on the study questionnaires to the benefit or harm gained from the doctor’s appointment as assessed by the doctor, parent, child and researchers (Fig. 2). Under the current legislation in Finland, omitting some of the school doctor’s obligatory health checks and comparing groups of children who undergo a health check to children who do not undergo one cannot be done. To circumvent this problem, the school doctor’s check is arranged to all children, as usual, without knowledge of the content of the previously filled study questionnaires. At one year after the health check, the implementation of the doctors’ interventions and follow-up plans will be analyzed.

### Study participants

This study is conducted in primary schools of four urban municipalities in Southern Finland. One municipality (Helsinki) has a system of exclusive school doctor services (i.e. physicians working solely as school doctors), whereas in three municipalities (Tampere, Kerava and Kirkkonummi) physicians also serve other population groups. All the children who met the inclusion criteria were given a computer generated random number by the study nurse. The first 30 children in each school and their parents were asked to participate. If more than 5 families did not participate, more children were recruited from the random order list. From each school, we will recruit at least 25 randomly selected children from grade 1 and 25 children from grade 5. Thus, the number of recruited children will be 1050 from 21 schools.

**Fig. 3:**
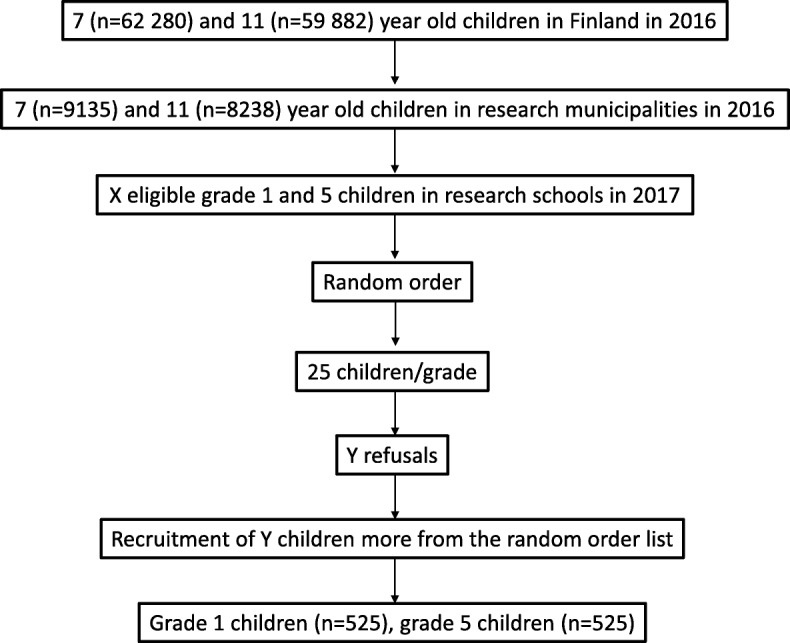
Flow chart of population-based recruitment

**Fig. 4:**
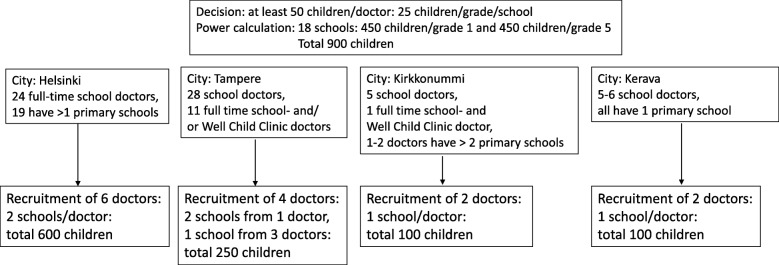
Recruitment of doctors in 4 municipalities. In Helsinki all school doctors who gave consent were chosen. The school doctors chose 2 different schools from different socioeconomic areas of Helsinki if possible. In Tampere, Kirkkonummi and Kerava the chief physician chose school doctors who had different education and experience of working as a school doctor and schools from different socioeconomic areas of the municipality. The school nurses and teachers were chosen according to the school doctors’ schools. In Helsinki 2 nurses refused to participate and the doctor chose another school instead

Fourteen doctors are participating in the study. Figure 3 depicts the flow chart of population-based recruitment and Fig. 4 outlines the recruitment of doctors.

Exclusion criteria are children studying mainly in special education groups and the need of an interpreter. The study is limited to Finnish speaking schools.

### Data collection procedure

Prior to the beginning of children’s recruitment, informed consent was obtained from all participating school nurses, teachers and doctors. School doctors filled a short form regarding their education and work experience. KN signed the school nurses’ and doctors’ consent forms. The teachers consent forms were signed by KN or the research assistant.

School nurses fill in study questionnaires (nurse’s questionnaire, NQ) after they have performed their part of the children’s extensive health check. The nurses store the NQs separate from the regular patient files. Before the school doctors’ health checks, nurses check the NQs to make sure that the information is up to date and mail the families study information, consent forms and study questionnaires (parent’s questionnaire, PQ). The participating school nurses sign the parents’ and children’s consent forms. The parents and children return their consent forms and the PQs to a sealed box in the waiting room before the doctor’s appointment. If the family has forgotten or lost their forms and PQs, they may fill them just before the doctor’s appointment. The nurses also deliver study questionnaires to the teachers (teacher’s questionnaire, TQ) who return them to the researchers by mail.

School doctors perform the health checks as usual without knowledge of the content of the study questionnaires (NQ, PQ, TQ). After each health check, doctors fill an electronic report including given instructions, significant discussions and follow-up plans (Fig. 1, box Interventions). In addition, school doctors report their estimate of the benefit or harm of the medical appointment based on the criteria defined by the researchers. Parents and children fill patient-reported experience measures (PREMs) regarding the benefit or harm of the doctor’s appointment.

School nurses inform the researchers about families that do not wish to participate and the reason for not participating (no consent, has moved from area/school, non-attendance at appointment). If the family doesn’t consent to the study, the nurse’s and teacher’s questionnaires are destroyed.

**Fig. 5:**
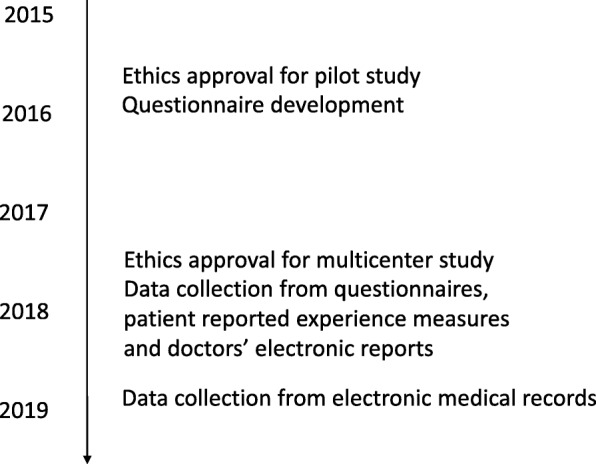
Timeline of the study

Data will be collected from doctors, children, parents/carers, teachers and nurses in 2017–2018 (Fig. 5). Date of enrolment of the first participant was August 22nd, 2017. To evaluate the implementation of the doctors’ care/treatment plans, the children’s electronic medical records will be assessed after one year’s follow-up. The exact study plan for this remains to be finalized. Additional file 1 outlines the Information sources and Data collection objects.

### Study questionnaires

The study questionnaires address parents’ (PQ), school nurses’ (NQ) and teachers’ (TQ) concerns regarding each child’s physical and mental health and the wellbeing of the whole family (Table 3). See Additional files 2, 3, 4 for the English versions of the study questionnaires. The questions were partly chosen from the Strengths and Difficulties Questionnaire (SDQ) which is a validated screening method for children’s psychiatric disorders [9]. One of the questions of the SDQ is almost as reliable as the complete SDQ [10–12]. That question evaluates whether the child has difficulties in one or more of the following areas: emotions, concentration, behavior, or being able to get on with other people. It was divided into four questions in the study questionnaires. Additional questions, regarding the child’s growth, physical well-being, eating, sleeping, learning, school absenteeism, and the well-being of the whole family, were also included based on previous evidence [13–18] and clinical knowledge of the research group and piloted.

### Study questionnaire, PREM and doctors’ electronic report development

Study questionnaires for parents, nurses and teachers and a doctor’s electronic report were developed between June and August 2015. The feasibility of the questionnaires and the electronic report was tested in a pilot study between November 2015 and May 2016 by one school doctor (KN) in three primary schools in Helsinki. Of the 147 families contacted, 132 (90%) chose to participate. In addition, three nurses and 15 teachers participated in this phase. Based on the pilot study, the questionnaires were slightly modified to better suit their purpose.

**Fig. 6:**
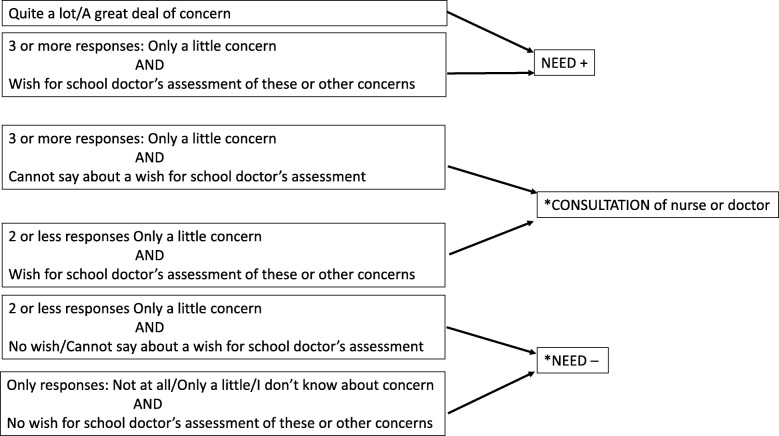
Categorization of parent’s and nurse’s study questionnaire responses. *The free description of the concern can alter the categorization to 1) NEED+ for school doctor’s health check: If there is concern such as parenthood or the relationship between parent and child, sleep problems, behavior problems in the class, recurrent joint pain/headaches, heel pain, acne, a mole, 2) CONSULTATION, (**a**) of doctor by nurse if the nurse has only little concern about growth or posture and a wish for school doctor’s assessment, (**b**) of nurse by parent if the parent has concern about: growth but the nurse is not concerned about it, the amount of sleeping, growth pain

**Fig. 7:**
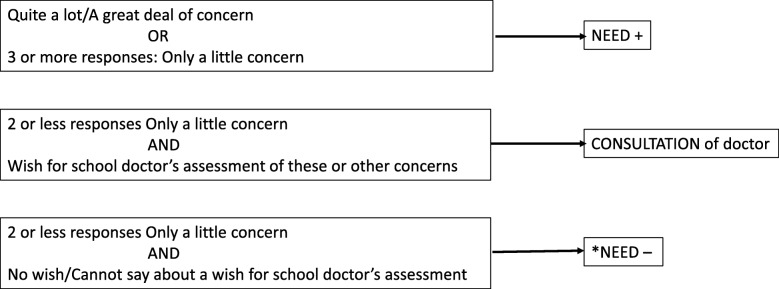
Categorization of teacher’s study questionnaire responses. *The free description of the concern can alter the categorization to NEED+ for school doctor’s health check: If there is concern such as parenthood or the relationship between parent and child, sleep problems, behavior problems in the class, recurrent joint pain/headaches, heel pain, acne, a mole

Two researchers (KN and EH) reviewed the questionnaires. The research group categorized the responses into three groups: 1) “Needs doctor’s health check” 2) “No need for doctor’s health check” and 3) “Consultation of the nurse by parent or consultation of the doctor by nurse or teacher may be sufficient”. The free description of the concern can alter the categorization (Figs. 6 and 7). Depending on the outcome of the consultation, a nurse’s or doctor’s appointment would be arranged for the child and parent if needed.

Based on the experience gained in the pilot study the criteria for the doctors’ and researchers’ evaluation of benefit and harm were defined (Table 2). Space was also provided for free responses of benefit or harm for all respondents.

**Table 2:** Criteria of benefit and harm and Patient reported experience measures (PREMs)

	Response options for doctors and researchers^b^							
	Benefit				Harm			
	A great deal	Quite a lot	Only a little	No benefit or harm	Only a little	Quite a lot	A great deal	
Criteria for doctors and researchers								
1. Significant discussion^a^ or other intervention that presumably reduces other health care use	x							
2. Need to contact child welfare	x							
3. Some referrals to secondary care	x	x						
4. Doctor’s role irreplaceable by nurse		x						
5. Presumably reduced concern^b^		x						
6. Some significant discussions^b^		x						
7. Nurse could have replaced the doctor			x					
8. No significant harm as consequence of unhandled concerns			x					
9. Suspicion that interaction failed or suspicion of no progress in care					x			
10. The interaction failed or here was no progress in care						x		
11. Suspicion of negative PREM or refusal of school doctor services in the future and no progress in care							x	
PREM question								
How much benefit or harm did you perceive from the school doctor’s health check?								I don’t know
Response options for parents	x	x	x	x	x	x	x	x
Response options for children		x	x	x	x	x		

^a^Relates to a different subject than an instruction, a prescription or a referral;

Additional criteria:

The child’s or parent’s concern reduced significantly or their resources strengthened or

The child or parent realized something new that improves their well-being or

The child or parent made a decision towards a healthier lifestyle

^b^The researchers take the PREMs into account when considering the value of discussions

If the parent’s PREM is between “Only a little benefit” and “A great deal of harm”, the value of discussions cannot be higher than “Only a little benefit”

The child’s PREM will be analyzed separately in a similar fashion

All responses of harm will be analyzed individually

The research group developed patient-reported experience measures (PREMs) for the parent and child to enable the evaluation of the parents’ and child’s perception of benefit or harm of the doctor’s health check. The response options for parents were the same as for doctors and researchers but without previously defined criteria (Table 2). In addition to a verbal scale, visual analogues in the form of facial expressions were included for children. Borg gave permission to use the same facial expression models that she used for the “Child’s self-evaluation enquiry on emotional well-being” [12]. See Additional file 5 and Additional file 6 for the English versions of the PREMs.

### Questionnaire delivery

The pilot study demonstrated a need for thorough instructions to all participating families and professionals. If the study questionnaire is completed too early, the likelihood of changes in the child’s health and life situation increases. The nurses were therefore instructed to check the previously filled NQ and to deliver the TQ just prior to sending the invitation letter to the parents. The teachers were instructed to return the TQs within one week.

Detailed written instructions and training sessions were arranged for school nurses (1.5 h), teachers (15 min) and school doctors (1–1.5 h) prior to the multicenter study. KN is available to answer questions from the participating nurses, school doctors, teachers and parents during the whole data collection period via phone or e-mail.

### Measures

#### Primary outcomes

The primary outcome measures are 1) the need for a school doctor’s health check and 2) the benefit/harm of school doctors’ routine health checks.

The need for the doctor’s health check is determined based on the parents’, teacher’s and nurse’s study questionnaires (Table 3). The responses will be categorized into three groups: 1) “Needs doctor’s health check” 2) “No need for doctor’s health check” and 3) “Consultation of the nurse or doctor may be sufficient” (Figs. 6 and 7).

**Table 3:** Areas of concern in the study questionnaires according to respondent

Areas of concern ^a^	Parent	Nurse	Teacher	
Child’s growth	x	x		
physical symptom(s)^b^	x	x	x	
hearing		x		
school absenteeism	x		x	
learning	x		x	
concentration	x	x	x	
behavior	x	x	x	
emotions	x	x	x	
getting on with others	x	x	x	
eating	x	x		
sleeping	x	x	x	
Wellbeing of family	x	x	x	
Free description of concern	x	x	x	
Wish for school doctor’s assessment of these or other concerns^c^	x	x	x	

^a^Response options for each area of concern on a five point Likert scale:

0 = Not at all, 1 = Only a little, 2 = Quite a lot, 3 = A great deal, 4 = I don’t know

^b^Specified in parent’s questionnaire: recurrent pain, prolonged complaints, skin symptoms,undescended testes

^c^Response options on a three-point scale: 2 = yes, 1 = I don’t know, 0 = no

Possible benefit and harm of a school doctor’s health check will be evaluated separately based on 1) the school doctors’ interventions and reports of benefit or harm and 2) the PREMs (Table 2).

#### Secondary outcomes

The secondary outcome measures are 1) the interventions and follow-up plans arising from school doctors’ routine health checks and 2) the implementations of measures from the doctor’s electronic report after a follow-up period of 12 months (Fig. 1, box Interventions). The implementation of the measures will be categorized into three groups (yes, no, information not accessible).

### Statistical methods

Sample size was calculated to detect 20% difference (25% vs 45%) in the benefit between children who need and children who do not need a doctor’s health check. Based on the pilot study, 25% of children who did not have a need for a doctor’s health check could benefit from one. To account for the clustered nature of the data, an intra-cluster (intra-school) correlation coefficient (ICC) of 0.06 was assumed. From each school, 25 children will participate from grade 1 and 25 children from grade 5. The required sample size is 450 children from both grades. Allowing for about 15% of missing data regarding the need for a health check and/or PREMs, the number of recruited children will be altogether 1050 from 21 schools.

Frequencies, percentages, means (with standard deviations; or in case of skewed distribution, medians with interquartile ranges) will be used as descriptive statistics.

Intrarater and interrater reliability will be assessed. KN will evaluate the need for the doctor’s health check and the benefit or harm of the doctor’s health check of each child and 200 randomly selected cases for intrarater reliability. To assess interrater reliability, SK will evaluate the data of 200 randomly selected children. SK will repeat the evaluation of the same 200 children to assess her intrarater reliability. Any discrepancies will be resolved using a third researcher.

The need for a doctor’s appointment and the benefit gained from it from the different people’s point of view will be compared. Researchers are blinded to study questionnaire responses when analyzing the benefit of the doctor’s appointment.

Multilevel logistic regression analyses will be conducted to account for the effect of covariates (e.g. doctors’ work experience and differences between municipalities). Analyses will be done separately for children from grade 1 and grade 5.

## Discussion

This is a multicenter study assessing the potential benefits or harm of school doctors’ routine health checks and the use of questionnaires to identify children who would be likely to benefit most from a school doctor’s health check. The study aims to evaluate whether school doctors should routinely check all children at ages of seven and eleven, as is currently mandated by the law, or only the ones that adults are concerned about. If the need for a doctor’s health check, as evaluated from the study questionnaires, predicts the researcher-evaluated benefit gained from the health check the questionnaires may be suitable for screening in the future. At one year after the health check, the implementation of the school doctors’ interventions and follow-up plans will be analyzed, which enables us to evaluate the effects of school doctors’ work.

The strengths of the study include the usability and generalizability of the results in several ways: 1) The study is conducted in a normal clinical setting of the children’s regular health checks; 2) The doctors performing the health checks are blinded to the answers of the study questionnaires; and 3) The study includes schools and professionals from different municipalities and socioeconomic areas. The multi-informant approach may be helpful in identifying the need for support for both children and their families. A limitation of the study is that the developed questionnaires have not been widely tested. It is possible that the questionnaires function best in the Finnish school healthcare system since the organizational models of school health service systems differ greatly even within Europe [2]. Additional limitations are that children studying mainly in special education groups and children whose parents need an interpreter were left out. According to clinical knowledge, the school doctor’s assessment is frequently beneficial if the child’s special education needs have not been assessed in a specialist setting or if the child has not undergone a medical examination related to a new immigrant status.

The current Finnish school healthcare system offers equal service for all children in certain grades. The obligation to double check the children by both a nurse and a doctor leads to a situation in which most of the school doctor resources are allocated to health checks of asymptomatic children whom no-one is concerned about. Children in other year levels than grades 1, 5 and 8 receive very limited school doctor services. Many children with special needs, often related to social inequities, mental health and lifestyle related problems, could benefit from the school doctor’s attention earlier and more often than is currently recognized and offered. School doctors have little time for multidisciplinary work in student welfare groups and for cooperation with family counseling, child welfare and secondary care. This can result in inefficient care and referrals of patients from one unit to another. A structured screening method that would be available at any time of the year would increase the timeliness of the school doctor’s health check. If the finding of this study is that recognizing children most likely to benefit from school doctors’ appointments is impossible using the developed screening method, we have provided scientific evidence to support the current practice of school doctors’ routine health checks in Finland. However, more school doctor resources should be provided to school health care to ensure timely access to school doctors’ evaluation in case of need regardless of school grade.

The study will increase our knowledge of the benefits or harm of school doctors’ routine health checks and assess the developed screening method. We hypothesize that targeting the doctors’ health checks to the children in greatest need would increase the quality of school health services.

## Additional files

Additional file 1: Information sources and data collection objects. (DOCX 71 kb)

Additional file 2: Form for identifying concerns (Parent’s Questionnaire). (PDF 43 kb)

Additional file 3: Form for identifying concerns (Nurse’s Questionnaire). (PDF 43 kb)

Additional file 4: Form for identifying concerns (Teacher’s Questionnaire). (PDF 43 kb)

Additional file 5: Feedback form (Parent’s patient-reported experience measure). (PDF 46 kb)

Additional file 6: Feedback form (Child’s patient-reported experience measure). (PDF 42 kb)

## Abbreviations

NQ: Nurse’s Questionnaire
PQ: Parent’s Questionnaire
PREM: Patient-reported experience measure
SDQ: Strengths and Difficulties Questionnaire
TQ: Teacher’s Questionnaire
