# Supplementary material for: Who benefit from school doctors’ health checks: a prospective study of a screening method
Source: BMC Health Serv Res. 2018 Jun 27;18:501. doi: 10.1186/s12913-018-3295-3 (PMC6020452; doi:10.1186/s12913-018-3295-3)
Supplement: Supplementary file 2 — Form for identifying concerns (Parent’s Questionnaire). (PDF 43 kb) [file 12913_2018_3295_MOESM2_ESM.pdf]

FORM FOR IDENTIFYING CONCERNS **patient number**
To be filled in by parents

For research purposes 2017-2018

	**Are you concerned about the child's** (Please check the most suitable option)	**Not at all**	**Only a little**	**Quite a lot**	**A great deal**	**I don't know**
1	growth or some physical symptom (recurrent pain, prolonged complaints, skin symptoms, undescended testes)					
2	school absenteeism					
3	learning					
4	concentration					
5	behavior					
6	emotions					
7	ability to get on with others					
8	eating					
9	sleeping					
10	the well-being of a family member or the whole family					

Do you wish to speak with the school doctor about these concerns or some other concern related to the child's well-being?

**Yes**	**No**	**I don't know**

Please describe your concern in your own words (if you wish, you can continue on the other side of the page)

____

____

____

Child's name and class ____________________________

Parent's name ____________________________

Date Parent's signature

__________ ____________________________
