# Supplementary material for: Who benefit from school doctors’ health checks: a prospective study of a screening method
Source: BMC Health Serv Res. 2018 Jun 27;18:501. doi: 10.1186/s12913-018-3295-3 (PMC6020452; doi:10.1186/s12913-018-3295-3)
Supplement: Supplementary file 3 — Form for identifying concerns (Nurse’s Questionnaire). (PDF 43 kb) [file 12913_2018_3295_MOESM3_ESM.pdf]

FORM FOR IDENTIFYING CONCERNS **patient number**

To be filled in by school nurse

For research purposes 2017-2018

	**Are you concerned about the student's** (Please check the most suitable option)	**Not at all**	**Only a little**	**Quite a lot**	**A great deal**	**I don't know**
1	growth					
2	hearing					
3	some physical symptom					
4	concentration					
5	behavior					
6	emotions					
7	ability to get on with others					
8	eating					
9	sleeping					
10	the well-being of a family member or the whole family					

Do you wish the school doctor to address these concerns or some other concern related to the well-being of the student?

**Yes**	**No**	**I don't know**

Please describe your concern in your own words (if you wish, you can continue on the other side of the page)

____

____

Student's name and class

____

School nurse's name ______________________________

Date

____

School nurse's signature

____
