# Supplementary material for: Who benefit from school doctors’ health checks: a prospective study of a screening method
Source: BMC Health Serv Res. 2018 Jun 27;18:501. doi: 10.1186/s12913-018-3295-3 (PMC6020452; doi:10.1186/s12913-018-3295-3)
Supplement: Supplementary file 5 — Feedback form (Parent’s patient-reported experience measure). (PDF 46 kb) [file 12913_2018_3295_MOESM5_ESM.pdf]

**FEEDBACK FORM** **patient number**
**To be filled in by the parent** *For research purposes 2017-2018*

**How beneficial or harmful do you think the school doctor's examination was?**

**(**Please check the part that you think best describes the benefit or harm)

| A great deal of benefit | Quite a lot of benefit | Only a little benefit | No benefit or harm | Only a little harm | Quite a lot of harm | A great deal of harm | I don't know |
|---|---|---|---|---|---|---|---|
| | | | | | | | |

**Please explain your answer**

____________________________________________________________

____________________________________________________________

____________________________________________________________

Student's name ________________________________

Student's date of birth ____________________

Parent's name ________________________________

Date ______________

Parent's signature ________________________________
