# Supplementary material for: Who benefit from school doctors’ health checks: a prospective study of a screening method
Source: BMC Health Serv Res. 2018 Jun 27;18:501. doi: 10.1186/s12913-018-3295-3 (PMC6020452; doi:10.1186/s12913-018-3295-3)
Supplement: Supplementary file 6 — Feedback form (Child’s patient-reported experience measure). (PDF 42 kb) [file 12913_2018_3295_MOESM6_ESM.pdf]

FEEDBACK FORM — ***patient number***

To be filled in by the student — *For research purposes 2017-2018*

How beneficial or harmful do you think the physician's examination was?

Please put a cross (x) in the box under the picture

**Quite a lot of benefit**	**Only a little benefit**	**No benefit or harm**	**Only a little harm**	**Quite a lot of harm**
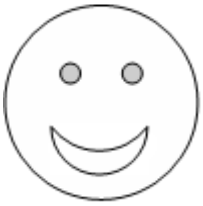	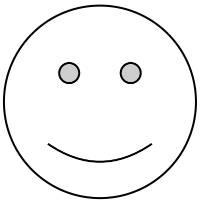	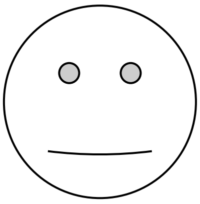	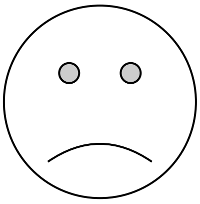	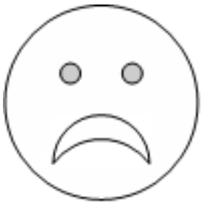

Please explain your answer:

____

____

____

Student's name

____

Student's date of birth

____

Date

____
