# Supplementary material for: Who benefit from school doctors’ health checks: a prospective study of a screening method
Source: BMC Health Serv Res. 2018 Jun 27;18:501. doi: 10.1186/s12913-018-3295-3 (PMC6020452; doi:10.1186/s12913-018-3295-3)
Supplement: Supplementary file 6 [file 12913_2018_3295_MOESM1_ESM.docx]

Additional file 1 Information sources and data collection objects

	Data collection object					
	child, grade1	child grade5	paren t	nurs e	teache r	docto r
**Information source**						
Doctors form: education and work experience						x
Parent's questionnaire			x			
Nurse's questionnaire				x		
Teacher's questionnaire					x	
Doctor's electronic report						x
